# To Swim or Not to Swim: Potential Transmission of *Balaenophilus manatorum* (Copepoda: Harpacticoida) in Marine Turtles

**DOI:** 10.1371/journal.pone.0170789

**Published:** 2017-01-23

**Authors:** Francesc Domènech, Jesús Tomás, José Luis Crespo-Picazo, Daniel García-Párraga, Juan Antonio Raga, Francisco Javier Aznar

**Affiliations:** 1 Marine Zoology Unit, Cavanilles Institute of Biodiversity and Evolutionary Biology, University of Valencia, Valencia, Spain; 2 Veterinary Services, Biology Department, Oceanogràfic, Valencia, Spain; University of Connecticut, UNITED STATES

## Abstract

Species of *Balaenophilus* are the only harpacticoid copepods that exhibit a widespread, obligate association with vertebrates, i.e., *B*. *unisetus* with whales and *B*. *manatorum* with marine turtles and manatees. In the western Mediterranean, juveniles of the loggerhead sea turtle, *Caretta caretta* are the only available hosts for *B*. *manatorum*, which has been found occurring at high prevalence (>80%) on them. A key question is how these epibionts are transmitted from host to host. We investigated this issue based on experiments with live specimens of *B*. *manatorum* that were cultured with turtle skin. Specimens were obtained from head-started hatchlings of *C*. *caretta* from the western Mediterranean. Hatched nauplii crawled only on rough substrates and lacked the ability to swim. Only copepodites IV and V, and adults, were able to perform directional swimming. Legs 2, 3 and 4 played a major role in swimming and were only well-developed in these stages. Nauplii reared in wells with turtle skin readily fed on this item. Late copepodites and adults also fed on turtle skin but did not consume other potential food items such as fish skin, baleen plates or planktonic algae. Evidences suggest that the transmission of *B*. *manatorum* should rely on hosts’ bodily contacts and/or swimming of late developmental stages between spatially close hosts. The possibility of long-ranged dispersal is unlikely for two reasons. First, all developmental stages seem to depend on turtle skin as a food resource. Second, the average clutch size of ovigerous females was small (< 70 eggs) for free-living phases to successfully contact turtles that occur at very low densities (< 0.6 turtles·km^−2^) in the western Mediterranean. The high prevalence of *B*. *manatorum* in loggerhead turtles in this area raises the question whether these turtles have contacts, or tend to closely aggregate, more than is currently believed.

## Introduction

Harpacticoid copepods are small crustaceans (range: 0.5–2.5 mm long) that inhabit most aquatic environments commonly as part of meiofauna [1] [2]. Four species, however, have been reported as being associated with vertebrates. *Neoscutellidium yeatmani* was found in the gills of the Antarctic fish *Lycodichthys dearborni* [3]. Although *N*. *yeatmani* exhibited apparent modifications for a parasitic life, this species has not been reported again since then. *Harpacticus pulex* was found associated, on a single occasion, with ulcerated skin areas on single individuals of the bottlenose dolphin, *Tursiops truncatus*, and the manatee, *Trichechus manatus*, in captivity [4]. Species of *Harpacticus*, including *H*. *pulex*, are typically associated with intertidal algae [5], thus raising the possibility that the occurrence of *H*. *pulex* in marine mammals was exceptional, and related to the hosts’ confinement conditions [4]. Finally, species of *Balaenophilus* exhibit a widespread association with vertebrates. *Balaenophilus unisetus* has been reported on the baleen plates of whales of the genus *Balaenoptera* in both the Atlantic and the Pacific Oceans [6] [7] [8]. A second species, *B*. *manatorum*, occurs on the skin of marine turtles and manatees, including wild or captive loggerhead sea turtles, *Caretta caretta*, from the western Mediterranean and Japan [9] [10] [11] [12], olive ridleys, *Lepidochelys olivacea*, and green turtles, *Chelonia mydas*, in the Pacific coast of Mexico [13] [14], and West Indian manatees, *Trichechus manatus*, in the western Caribbean [15] [16].

All developmental stages of species of *Balaenophilus* present modified appendages for clasping the skin of their hosts. Nauplii have antennae and mandibles with strong claws, whereas copepodites and adults have the maxilliped and the first leg modified as strongly developed clasping organs [9]. These observations suggest that species of *Balaenophilus* have an obligate association with their vertebrate hosts [12]. The mouthparts appear to be adapted for scraping surfaces [6] [9] [10]. In the case of *B*. *unisetus*, individuals ingest baleen tissue [10], perhaps along with algal-diatom film associated with it [6], with no apparent harm to the host. However, in loggerhead marine turtles, *B*. *manatorum* appears to ingest host skin, eroding the tissue and sometimes causing lesions [9] [10], whereas in manatees no apparent lesions have been observed [16]. Regardless of the impact on their hosts, species of *Balaenophilus* seem to be adapted to exploit keratin-rich microhabitats [10].

An interesting, unanswered question is how species of *Balaenophilus* are transmitted from host to host. Ogawa (1997) [9] speculated that these copepods should spend their entire life on their host based on the apparent non-swimming morphology of the nauplii. However, both copepodites and adults, although adapted to clasping the host, also have appendages (particularly legs 2–4) that could be involved in swimming (see [9]; [11]). Therefore, two non-mutually exclusive strategies for transmission could be hypothesized, i.e., bodily contact between hosts, as in some ectoparasitic crustaceans (e.g., whale-lice, which cannot swim; see [17]; [18]), or through free-living dispersal stages (i.e., copepodites and/or adults). In fact, the vast majority of crustacean symbionts include free-living stages (nauplii and/or 1^st^ copepodites) in their life cycles [19].

The hypothesis of transmission by contact is plausible in the case of *B*. *unisetus* in whales, and *B*. *manatorum* in manatees, because these social mammals engage in frequent interactions [20] [21]. However, marine turtles are solitary reptiles even when individuals share the same foraging or breeding areas [22] [23], with interactions between individuals being anecdotal except during mating [24] [25]. This raises the possibility that copepodites and/or adults of *B*. *manatorum* also function as a dispersal free-living phase in this case.

In the present study we investigated the transmission of *B*. *manatorum* based on observations and experiments with live specimens obtained from loggerhead marine turtles and cultured with turtle skin. First, we gathered information on infection parameters and fecundity of gravid females of *B*. *manatorum*. Second, we analyzed the swimming performance of all developmental stages to examine the ability of each stage to use swimming as a dispersal mechanism. Third, we carried out feeding trials to investigate when individuals of *B*. *manatorum* start to feed, and whether they rely on turtle tissue and/or other items as a food resource. Feeding trials can give insight as to whether individuals could survive as plankton feeders or by exploiting other hosts, e.g., fish.

## Materials and Methods

### Ethics statement

Loggerhead sea turtles’ hatchlings examined in this study were reared in captivity at the Sea Turtle Rescue Center (ARCA del Mar) located at the Oceanogràfic aquarium of Valencia (Spain). Hatchlings came from a single nest that was found at the end of June, 2014, in a highly touristic and crowded beach in SE Spain. The clutch was carefully removed and relocated in a protected beach. Eggs hatched at the end of August, and a total of 87 hatchlings were brought to the marine rehabilitation center of the Oceanogràfic aquarium and were grown until they reached a pre-established weight (*ca*. 1 kg) to minimize predation after being released into the sea. This “head-starting” procedure is considered a useful conservation tool to increase survival of sea turtles during their first stages of development [26].

The protocol for copepod sampling was harmless for the infected hatchlings from which copepods were collected, and was always carried out under the supervision of the veterinary staff of the aquarium. Copepod individuals selected for the feeding trials were euthanized in hot 70% ethanol. The University of Valencia and the Oceanogràfic aquarium have a collaborative agreement with the Wildlife Service of the Valencian Regional Government, Spain, which is the official institution in charge of managing and protecting wildlife in the region. This institution approved the present study and certified that the entire process met appropriate ethical standards.

### Samples

Hatchlings were reared in a polypropylene cylindrical tank (26x10^3^ l), every one within individual cylindrical plastic-mesh floating cages (diameter: 50 cm; height: 50 cm; mesh: 1 cm). The use of cages allowed individual identification of turtles and also prevented bite injuries among them. Cages drifted on the surface of the tank so that they frequently contacted at random, sometimes forming aggregations. Water temperature was set at 25°C (±1) and full light spectrum was provided through hydrargyrum quartz iodide (HQI) lightning.

The facilities also kept injured wild marine turtles, bycaught or stranded, that had been rescued by the wildlife service. Although hatchlings were usually kept in their own tank, occasionally, and due to space constraints, hatchlings were maintained for several days within their floating cages in another tank where wild turtles on rehabilitation were also kept. Both tanks shared a filtration system composed of protein skimmers, ultraviolet light, biological filtration and ozone to maintain the quality of recirculating water.

At the beginning of this study in January 2015, a total of 57 hatchlings remained alive in the aquarium. All turtles were observed to have clinical signs consisting of acute rubbing behavior, anorexia, weight lost and lethargy. Upon examination, heavy infestations of *B*. *manatorum* (> 300 individuals per host) associated with severe erosion of skin were detected in affected hatchlings. A total of 14 hatchlings were too debilitated so they were excluded for this study and subject to immediate deparasitation. In the remaining 43 turtles, we gently rubbed the skin with a small brush while flushing seawater with a wash bottle over a 0.2 mm sieve to collect specimens of *B*. *manatorum* prior to do a deparasitizing treatment. Copepods from 37 turtles were separately collected, fixed and preserved in 70% ethanol for morphological studies. Individuals from the remaining 6 turtles were cultured under similar conditions as the turtles: *ca*. 700 specimens were kept in each of two 1000 ml cylindrical plastic containers with 0.05 mm filtered seawater at 25°C with gentle aeration, and fed *ad libitum* with skin flakes of loggerhead turtle hatchlings. These skin samples were collected from hatchlings of the same clutch that died at the rescue center before the infestation was detected. Photoperiod during the culture period was 12L:12D.

### Infection parameters

Individuals of *B*. *manatorum* were counted in 37 turtles. The 95% confidence interval (95% CI) for prevalence (i.e., percent infected turtles) was calculated with Sterne’s exact method [27], and for mean values of intensity (i.e., mean number of individuals per infected turtle) with the bias-corrected and accelerated bootstrap method using 20,000 replications [28]. Calculations were performed using the free software Quantitative Parasitology v.3 [29]. It is important to note that the hatchlings had been subject to a first treatment round against *B*. *manatorum* with freshwater a week before we counted them.

From the samples conserved in 70% ethanol we randomly selected 4 ovigerous females from 5 turtles (i.e., 20 specimens in total) to count the number of eggs and calculate egg diameter. Pairs of egg sacs were separated from each female under a magnifying glass (8-40x). In each of five females, one egg sac was photographed with a digital microscope color camera Leica DFC295 using Leica Application Suite (LAS), and diameters of five eggs from the sac (total n = 25) were measured from pictures using the software Image J 1.48v [30]. The egg sacs from the 20 ovigerous females were then dissected with fine forceps and the number of eggs counted. To calculate clutch size per female, the number of eggs from the two sacs were summed up.

### Transmission analysis

#### 1. Hatching and mobility of nauplii

Thirty-six ovigerous females were randomly selected from the culture of *B*. *manatorum* and individually separated in Petri dishes 40x12 mm with 3 ml of filtered seawater. Each Petri dish was checked every half hour until hatching of nauplii occurred. The hatching process was videotaped (n = 3) under a magnifying glass (20- 40x) with a digital microscope color camera Leica DFC295 operating at 25 frames per second and a resolution of 3.1 megapixels. After hatching, individual clutches (n = 5) were put in Petri dishes as described above and all nauplii (*ca*. 250 individuals in total) were examined every 6 h to record behavior until the last individual died.

#### 2. Morphology and swimming patterns of copepodites and adults

Thirty-four individuals (10 adult females, 10 adult males, 5 copepodite V, 3 copepodite IV, 3 copepodite III and 3 copepodite II) were randomly selected from the culture and separated individually in Petri dishes 150x25 mm with 8 ml of filtered seawater to videotape movements as described above. No live copepodite I individuals could be found in the culture. Movements of each individual were videotaped under a magnifying glass (8-10x) for 5–15 min. Videos were played in slow motion (25% of original speed) to describe swimming patterns of each individual. Description included the pattern of movement and a record of active appendages during swimming.

Forty individuals (6 adult males, 6 adult females, 5 copepodite V, 8 copepodite IV, 5 copepodite III, 5 copepodite II and 5 copepodite I) were randomly selected from the sample of individuals preserved in 70% ethanol to describe and measure the appendages. Each individual was put laterally under a magnifying glass (5-20x) and photographed as described above. Then, legs involved in generating thrust for swimming (i.e., 2, 3 and 4, see the Results) were separated individually using ultra fine forceps (tip dimensions: 0.01 x 0.005 mm) and photographed. Body length (from anterior end to end of caudal ramus) and legs 2, 3 and 4 (when developed) were measured using Image J 1.48v. In legs, only the exopod was measured, from the anterior edge of the basis to the posterior edge of the last leg segment. For each leg, all exopod setae were also measured from basis to anterior tip, and values were averaged. Setal formulae [31] of legs 2, 3 and 4 for each developmental stage were recorded.

### Feeding trials

Thirty-six ovigerous females were randomly selected from the culture and individually separated in Petri dishes of 40x12 mm with 3 ml of 0.05 mm filtered seawater to obtain nauplii. Females were removed immediately after the eggs hatched. A total of 7 nauplii from each of 4 randomly selected Petri dishes (total n = 28) were euthanized in hot 70% ethanol, individually mounted on cavity well slides and observed under light microscope (40x) to check for gut contents. Then, in 31 Petri dishes (average no. nauplii: 53.9 ± 9.7) a few skin flakes from loggerhead sea turtle hatchlings were added, whereas in 5 Petri dishes (average no. nauplii: 55.8 ± 5.9) no skin was provided. After 36 h, a total of 18 nauplii from five Petri dishes with turtle skin were mounted on cavity well slides and observed under light microscope (40x) to check for gut contents. The remaining samples were checked every 6 h until the last individual of the clutch died. A Mann-Whitney test was used to compare the survival time between clutches with, and without, turtle skin.

In the feeding trials involving copepodites and adults, we ensured that the selected specimens had no gut contents prior to the beginning of experiments. To do this, we selected 10 copepodites and adults from the culture with food pellets in the gut (observed under stereomicroscope at 20x). After 24 h, these individuals were euthanized in hot 70% ethanol and mounted on cavity well slides; the gut was empty in all cases, indicating that starvation for 24 h ensured complete elimination of digested food. Then, fifty individuals (copepodite V and adults) were randomly selected from the culture and separated individually in 24-multiwell plates of 14x15 mm with 3 ml of filtered seawater. After 24 h, flakes of baleen plates from fin whale (*Balaenoptera physalus*), available from the Marine Zoology Unit Collection at the University of Valencia, were added to 13 wells; skin of blue whiting (*Micromesistius poutassou*) in 13 wells; 1 ml filtered seawater with the green alga *Tetraselmis* sp. (concentration: 80x10^4^ cel·ml^-1^) in 12 wells, and skin flakes of hatchling loggerhead sea turtle in 12 wells. *Tetraselmis* sp. is a typical prey used in experiments involving planktonic copepods [32]. All food items were supplied in excess to prevent starvation. Except *Tetraselmis* sp. (which exhibits a natural green color), food items were stained with Brilliant Blue FCF (E-133) during 24 h before supply. After 36 h, copepods were euthanized and observed under a magnifying glass (5-10x) or light microscope (10-40x) to examine gut contents.

## Results

### Infection parameters

**Fig 1:**
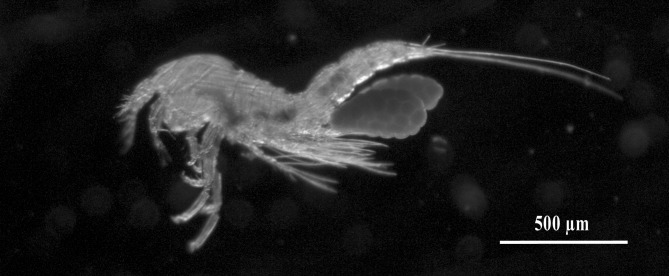
Ovigerous female of *Balaenophilus manatorum* collected from a hatchling of loggerhead sea turtle, *Caretta caretta*.

The percentage of turtles infected with *B*. *manatorum* was 100% (95% CI: 92.0–100.0) and mean intensity ± SD was 337.4 ± 212.9 (95% CI: 249.7–462.5) [range: 17–1423] copepods per turtle. Copepods occurred mainly on the ventral surface of the flippers, pectoral girdle, pericloacal skin and plastron sutures. Ovigerous females (n = 20) had a pair of egg sacs (Fig 1) with an average clutch size ± SD of 68.4 ± 8.7 (range: 53–92) eggs per female, and an average egg diameter ± SD of 52.5 ± 5.9 (range: 41–64) μm. In two ovigerous females, new egg sacs were observed 2–3 days after nauplii were hatched and the empty egg sacs released.

### Transmission analysis

**Fig 2:**
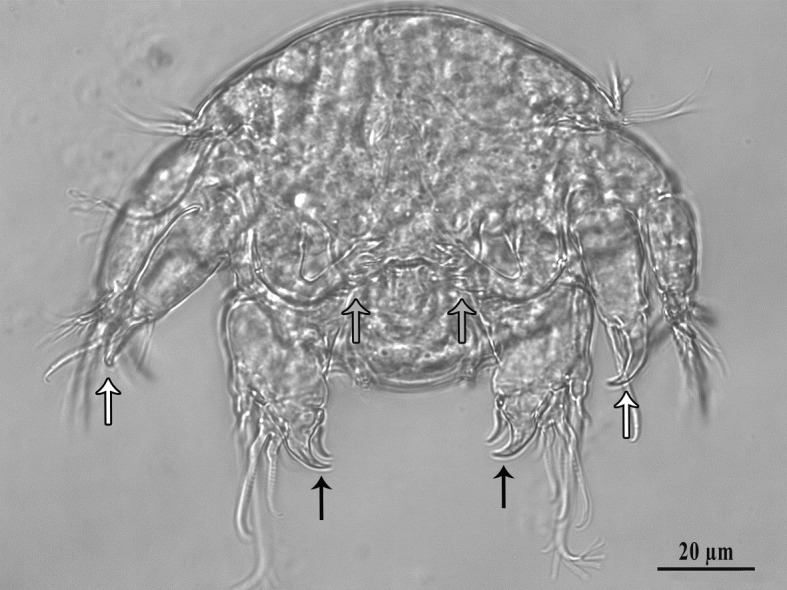
Nauplius of *Balaenophilus manatorum* collected from a hatchling of loggerhead sea turtle, *Caretta caretta*. Note appendages that are used to grasp on the host: antennae (black arrows) and mandibles (white arrows). Also note the dentate coxa of antennae (grey arrows) that functions as a food-processing appendage.

After hatching, the nauplii crawled over the female to reach the substrate immediately below it (S1 Video). Nauplii were able to crawl when they could grasp protrusions on rough substrates, or filamentous material, with the distal claws of appendages (Fig 2). However, in Petri dishes containing seawater only, nauplii remained for up to 4 days on the very same point where they had been left until they died. Nauplii exhibited no ability to swim. The duration of the naupliar phase could not be determined because all nauplii died before reaching the first copepodite phase.

**Fig 3:**
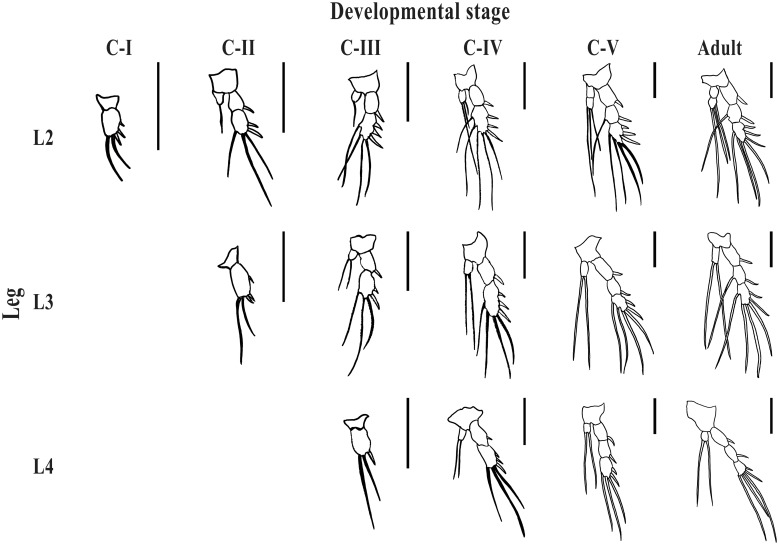
Morphology of legs 2 (L2), 3 (L3) and 4 (L4) of five copepodite stages (CI-CV) and adults of *Balaenophilus manatorum* collected from loggerhead sea turtles, *Caretta caretta* (Scale bar: 100 μm; see text for details).

**Fig 4:**
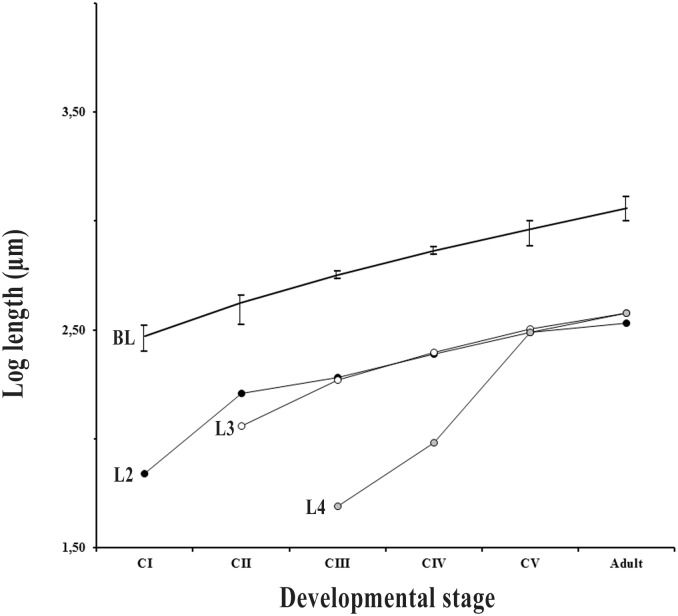
Measurements of body length (BL) and exopod of legs 2 (L2), 3 (L3) and 4 (L4) of five copepodite stages (CI-CV) and adults of *Balaenophilus manatorum* collected from hatchlings of loggerhead sea turtle, *Caretta caretta* (see text for details).

Morphometric and meristic features of legs 2, 3, and 4 for each copepodite stage and adult males and females are shown in Figs 3 and 4 and Table 1 (see also S1 Table). Copepodite I had leg 2 developed (only exopod), and it was comparatively shorter relative to body length than in later stages. Copepodite II had legs 2 and 3 (only exopod) developed, and leg 3 was relatively shorter than in later stages (Figs 3 and 4). Copepodite III had legs 2, 3 and 4 (only exopod) developed, and leg 4 was relatively shorter than in later stages. Copepodites IV, V and adults had legs 2, 3 and 4 with both exopod and endopod, but the exopod was 2-segmented in the copepodite IV and 3-segmented in later stages (Table 1). Also, leg 4 was relatively shorter in the copepodite IV than in later stages (Figs 3 and 4). The only meristic difference between copepodite V and adults was that in the second segment of the exopod of leg 3 there was a seta in the adult, but not in the copepodite V (Fig 3, Table 1).

**Table 1:** Meristic features of appendages of the five copepodite stages, and adult females and males, of *Balaenophilus manatorum* collected from hatchlings of loggerhead sea turtle, *Caretta caretta*. The number of segments in exopod and endopod are in parentheses, followed by the setal formula: Roman numbers stands for spines, and Arabic numbers for setae (see [29] for details). A brief account of swimming pattern is also provided.

			CI (n = 5)	CII (n = 5)	CIII (n = 5)	CIV (n = 8)	CV (n = 4)	Adult (n = 14)
**Setal Formula**	**L2**	**Exopod**	(1) III,2,0	(2) I-0; II,2,1	(2) I-0; III,2,2	(2) I-0; III,2,3	(3) I-0; I-1; III,2,2	(3) I-0; I-1; III,2,2
		**Endopod**	-	(1) 0,1,0	(1) 0,1,0	(1) 0,3,0	(2) 0–0; 0,3,0	(2) 0–0; 0,3,0
	**L3**	**Exopod**	-	(1) III,2,0	(2) I-0; II,2,1	(2) I-0; III,2,2	(3) I-0; I-0; II,2,2	(3) I-0; I-1; II,2,2
		**Endopod**	-	-	(1) 0,2,0	(1) 0,2,0	(1) 0,2,0	(1) 0,2,0
	**L4**	**Exopod**	-	-	(1) I,2,0	(2) I-0; III,2,1	(3) I-0; I-0; II,2,1	(3) I-0; I-0; II,2,1
		**Endopod**	-	-	-	(1) 0,2,0	(1) 0,2,0	(1) 0,2,0
**Swimming pattern**			?	Circular/loops No net displacement	Erratic No net displacement	Directional Net displacement	Directional Net displacement	Directional Net displacement

In the culture containers, both copepodites and adults were always found under the layer of turtles’ skin flakes holding onto them. When forced to swim, legs 2, 3 and 4 (depending on the developmental stage), along with antennulae and antennae, performed backward strokes (S2 Video). It was not possible to collect live Copepodite I individuals. Copepodite II performed circular or figure-eight looping movements with no net displacement, whereas Copepodite III exhibited erratic swimming with little net displacement (S3 Video). Later stages were able to perform directional, rapid swimming (S3 Video).

### Feeding trials

**Fig 5:**
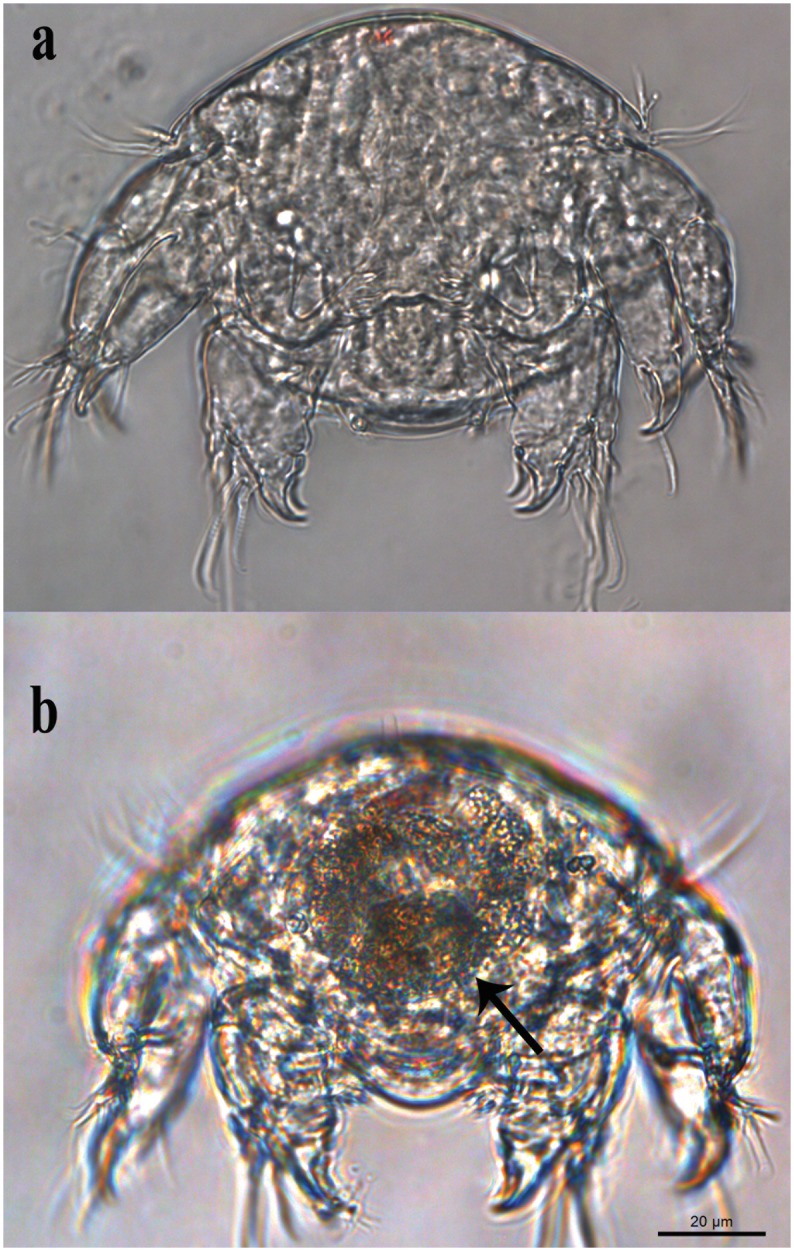
Feeding trials with nauplii of *Balaenophilus manatorum* collected from hatchlings of loggerhead sea turtle, *Caretta caretta*. (a) Nauplius immediately after hatching. (b) Nauplius after 36 h. Note skin flakes of turtle in the gut (arrows).

Recently hatched nauplii (n = 7) had no gut contents (Fig 5a) but, after 36 h in Petri dishes with skin flakes of turtles, all nauplii (n = 18) had brownish gut contents (Fig 5b). The average survival duration ± SD (range) of the last nauplius in 31 clutches cultured with turtle skin flakes was 8.0 ± 7.0 (0–22) days. In contrast, the average survival duration ± SD (range) of the last nauplius in 5 clutches without turtle skin was 1.6 ± 2.0 (0–4) days (n = 5 clutches). The difference of survival time was significant (Mann-Whitney test, U = 32.5, p = 0.037).

**Fig 6:**
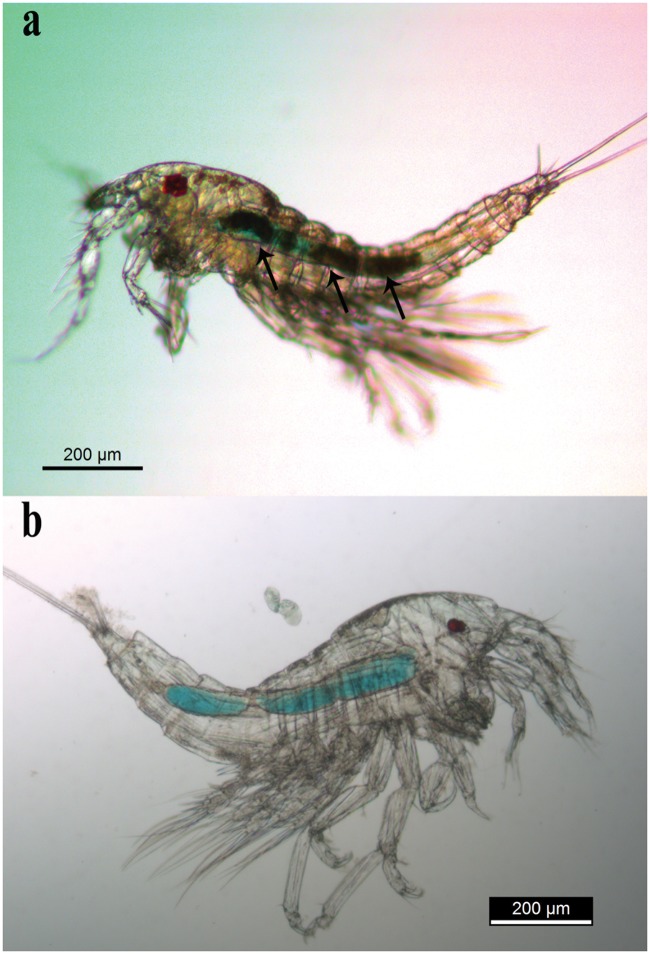
Feeding trials with copepodites and adults of *Balaenophilus manatorum* collected from hatchlings of loggerhead sea turtle, *Caretta caretta*. (a) Copepodite V with blue-dyed skin flakes of turtle in the gut (arrow). (b) Adult without solid food in the gut after 36 hours of exposure to blue-dyed flakes of baleen plate from a fin whale, *Balaenoptera physalus*.

All copepodite V and adult specimens that were put with blue-colored skin flakes of loggerhead sea turtle had solid blue gut contents after 36 h (Fig 6a). Individuals reared with *Tetraselmis* sp., baleen flakes or fish skin had no solid food in the gut after 36 h. Note, however, that water in wells became faintly stained in blue due to slight discoloration of stained food. The gut of copepods in wells with baleen flakes or fish skin was bluish (Fig 6b), indicating that they had swallowed water from the well.

## Discussion

All loggerhead sea turtle hatchlings examined in this study harbored nauplii, copepodites and adults (including gravid females) of *B*. *manatorum* indicating that hatchlings are suitable hosts for this copepod. It is also clear that turtles got the infection in the rearing facility because they were not exposed to wild conditions. Hatchlings were temporarily kept in tanks that also harbored rehabilitating juvenile turtles caught in the wild, and the filtration system was shared between all tanks. Thus, rehabilitating turtles most likely were the source of infection. In fact, after the epizootics, 8 wild turtles to be rehabilitated were examined for *B*. *manatorum* prior to entering the tank and 6 of them were positive for this copepod (unpub. data). Hatchlings could have become infected by direct contact or through the shared filtration system. Captivity surely enhanced opportunities for transmission between loggerhead sea turtle hatchlings, thus the infection spread rapidly. Furthermore, hatchlings have an underdeveloped immune system, which might have facilitated population growth of *B*. *manatorum*. Interestingly, the infection of *B*. *manatorum* that Ogawa (1997) [9] reported on a juvenile loggerhead sea turtle in Japan also occurred in a rearing facility but it was not possible to identify the source of infection.

Observations of the nauplii of *B*. *manatorum* clearly indicate that they can crawl by grasping protrusions on rough substrates, but they cannot swim. Inability of nauplii to swim is common among harpacticoids; only species of the Longipediidae and the Canuellidae (suborder Polyarthra), and perhaps some harpacticoids symbiotic on invertebrates, appear to have naupliar stages with good swimming abilities [1]. In contrast, copepodites and adults of *B*. *manatorum* were able to swim. However, swimming performance substantially improved during ontogeny. Copepodites II and III were unable to get net displacement in the water column, whereas later stages, particularly copepodites V and adults, performed fast, directional swimming. Although we did not carry out a detailed analysis of swimming performance, morphological data open the possibility that differences in swimming ability are related to the sequential acquisition of legs 2, 3 and 4; only animals having all these legs were able to swim directionally.

Results of feeding trials demonstrate, for the first time, that *B*. *manatorum* consume turtle skin. Nauplii consume it as soon as they hatch; in fact, the feeding apparatus of nauplii is adapted to scrapping surfaces [9], which would make sense for a crawling organism that hatches and feeds on its host. We cannot rule out that nauplii could use other food resources. For instance, in our experiment, nauplii reared in wells without turtle skin were exposed to water with organic particles < 0.05 mm, which could perhaps be swallowed by the animals (see below). However, these individuals had lower survival than nauplii feeding on turtle skin. On the other hand, copepodites or adults of *B*. *manatorum* are also adapted to scraping surfaces [9] [10], and results from feeding trials indicate that they did readily consume turtle skin, but not fish skin or baleen tissue, which are two potential alternative sources of animal epidermis or keratin, respectively. However, there is evidence that specimens reared in wells without turtle skin swallowed water, so we cannot rule out that they could derive some energy from organic particles < 0.05 mm suspended in water. But, contrary to typical planktonic copepods [33], individuals of *B*. *manatorum* did not consume *Tetraselmis* cells when these planktonic prey were available.

The above evidence sheds new light on several non-mutually exclusive transmission strategies of *B*. *manatorum*. In principle, all developmental stages could be transmitted through physical host-to-host contacts, similarly as human lice do [34]. However, the ability of directional swimming of late copepodites and adults would facilitate preferential transference of these stages. Interestingly, adults are also the most mobile stage in human lice, and experimental data have demonstrated that they are the most likely stage to initiate new infestations [34]. A second possibility is that transmission of *B*. *manatorum* occurs when hosts are spatially close to each other. Here, successful transmission would require the ability of the copepod to (1) detect approaching hosts and (2) perform directional swimming. Experimental data have shown that larvae of obligate commensal crustaceans specific to marine turtles or whales are able to recognize chemical cues emanated from their hosts (e.g., [35]) Thus, the possibility that late stages of *B*. *manatorum* could follow gradients of chemical substances or hydrodynamical signals over short distances to reach turtles cannot be rule out.

The last hypothesis is that long-range dispersal of *B*. *manatorum* would also be used as a transmission strategy. Accordingly, nauplii and early copepodite stages would act as passive pelagic drifters (see [36]; [37]), and/or late copepodites and adults as more active seekers of hosts. Dietary data, however, suggest that all stages appear to heavily rely on turtle skin as a food resource. The morphology of the feeding apparatus, as well as dietary experiments, would suggest that potential long-range dispersal would depend, at best, on food resources of poor quality, thus restricting the period for transmission. Interestingly, the vast majority of other harpacticoids with good swimming ability restrict their incursions to the planktonic realm to short periods (< 2 hours [37]), and only travel short distances (< 20 m [38]).

Reproductive data are compatible with the hypothesis that transmission of *B*. *manatorum* can hardly rely on long-range dispersal. In parasitic copepods, species with abundant or accessible hosts (i.e., those infecting schooling fish or benthic invertebrates) seem to exhibit low fecundity rates and larger eggs than those with sparsely distributed or inaccessible hosts ([39] [2] and references therein). At the family level, the data provided by Poulin (1995) [39] indicate clutch sizes ranging from 2 to 8967 eggs (median: 146) and egg size from 40 to 422 μm (median: 129) for a range of body sizes from 0.4 to 31.7 mm (median: 2.2). Figures obtained for *B*. *manatorum* (an average of 68 eggs of 52 μm for females 1.1 mm long) fall in the lower half of these ranges, even for harpacticoids (see [39]). Therefore, it would make little adaptive sense for *B*. *manatorum* to produce a few dozens of eggs for long-distance transmission through free-living stages among solitary hosts (i.e., marine turtles) found at very low densities. For instance, in the western Mediterranean, the population density of loggerhead sea turtles is just 0.59 turtles·km^−2^ [40].

The hypothesis that transmission of *B*. *manatorum* relies on bodily contacts between hosts and/or transference of late developmental stages through short distance swimming would be plausible only if hosts engage in frequent interactions and/or concentrate spatially at high densities. Sexual contacts in manatees and marine turtles, and mother-to-pup interactions in manatees, provide ample opportunities for direct transmission by contact. Also, aggregation of manatees in shallow coastal habitats for feeding [41], or adult marine turtles for mating or nesting [42] could facilitate transmission via short-range swimming of late copepodites and adults. However, the transmission scenario in the western Mediterranean is puzzling. This region is a feeding area for juveniles of loggerhead sea turtle that come from north Atlantic and eastern Mediterranean rookeries ([43] and references therein). Juvenile loggerhead sea turtles behave as solitary pelagic animals and the population density in the western Mediterranean is very low (see above). Yet the prevalence of *B*. *manatorum* in wild juvenile loggerhead sea turtles is 82.7% [10], and no other hosts are available for *B*. *manatorum* in this region. The question is, therefore, how so many juveniles become infected. Conventional wisdom indicates that, since the time of hatching, marine turtles have very limited contacts at sea with conspecifics [44]. However, epidemiological data from *B*. *manatorum* would suggest that juvenile loggerhead sea turtles could have had contacts, or tend to aggregate with conspecifics, other marine turtles, or even manatees, more than it is currently believed. Future studies should address this intriguing question.

## Supporting Information

S1 Table Measurements of body length and exopod of legs 2 (L2), 3 (L3) and 4 (L4), in μm (mean ± S.D. with range in parentheses), of five copepodite stages, and adult females and males, of *Balaenophilus manatorum* collected from hatchlings of loggerhead sea turtle, *Caretta caretta*. (DOCX)

S1 Video Hatchling process of nauplii of *Balaenophilus manatorum* collected from hatchlings of loggerhead sea turtle, *Caretta caretta*. (MP4)

S2 Video Movement of antennulae, antennae and legs 2, 3 and 4 during swimming in copepodite V and adult of *Balaenophilus manatorum* collected from hatchlings of loggerhead sea turtle, *Caretta caretta*. (MP4)

S3 Video Comparative swimming performance of copepodite III (erratic) and copepodite V (directional) of *Balaenophilus manatorum* collected from hatchlings of loggerhead sea turtle, *Caretta caretta*. (MP4)
