# Supplementary material for: To Swim or Not to Swim: Potential Transmission of Balaenophilus manatorum (Copepoda: Harpacticoida) in Marine Turtles
Source: PLoS One. 2017 Jan 23;12(1):e0170789. doi: 10.1371/journal.pone.0170789 (PMC5256940; doi:10.1371/journal.pone.0170789)
Supplement: S1 Table — (DOCX) [file pone.0170789.s001.docx]

|  |  | **CI (n = 5)** | **CII (n = 5)** | **CIII (n = 5)** | **CIV (n = 8)** | **CV (n = 4)** | **Adult (n = 14)** |
| --- | --- | --- | --- | --- | --- | --- | --- |
| **Body length** |  | 296 ± 32  (252 – 332) | 423 ± 51  (334 – 456) | 562 ± 23  (540 – 588) | 726 ± 22  (698 – 761) | 913 ± 99  (762 – 998) | 1143 ± 78  (1000 – 1291) |
| **Exopod length** | **L2** | 69 ± 24  (47 – 101) | 161 ± 6  (154 – 169) | 191 ± 11  (182 – 205) | 245 ± 11  (228 – 263) | 307 ± 9  (291 – 314) | 339 ± 19  (303 – 374) |
|  | **L3** | - | 115 ± 6  (108 – 124) | 186 ± 10  (170 – 196) | 250 ± 15  (216 – 267) | 318 ± 19  (287 – 333) | 376 ± 26  (318 – 414) |
|  | **L4** | - | - | 49 ± 3  (44 – 53) | 96 ± 5  (87 – 102) | 309 ± 11  (289 – 317) | 379 ± 24  (315 – 409) |

**S1 Table. Measurements of body length and exopod of legs 2 (L2), 3 (L3) and 4 (L4), in µm (mean ± S.D. with range in parentheses), of five copepodite stages, and adult females and males, of *Balaenophilus manatorum* collected from hatchlings of loggerhead sea turtle, *Caretta caretta*.**
